# Left ventricular support adjustment to aortic valve opening with analysis of exercise capacity

**DOI:** 10.1186/1749-8090-9-93

**Published:** 2014-05-20

**Authors:** Daniele Camboni, Tobias J Lange, Patrycja Ganslmeier, Stephan Hirt, Bernhard Flörchinger, York Zausig, Leopold Rupprecht, Michael Hilker, Christof Schmid

**Affiliations:** 1 Department of Cardiothoracic Surgery, University Medical Center Regensburg, Franz-Josef-Strauss-Allee 11, 93042 Regensburg, Germany; 2 Department of Internal Medicine II/Pneumology, University Medical Center, Regensburg, Germany; 3 Department of Anesthesiology, University Medical Center Regensburg, Regensburg, Germany

**Keywords:** Left ventricular assist device, Exercise testing, Right heart catheterization, Aortic valve

## Abstract

**Background:**

LVAD speed adjustment according to a functioning aortic valve has hypothetic advantages but could lead to submaximal support. The consequences of an open aortic valve policy on exercise capacity and hemodynamics have not yet been investigated systematically.

**Methods:**

Ambulatory patients under LVAD support (INCOR®, Berlin Heart, mean support time 465 ± 257 days, average flow 4.0 ± 0.3 L/min) adjusted to maintain a near normal aortic valve function underwent maximal cardiopulmonary exercise testing (CPET) and right heart catheterization (RHC) at rest and during constant work rate exercise (20 Watt).

**Results:**

Although patients (n = 8, mean age 45 ± 13 years) were in NYHA class 2, maximum work-load and peak oxygen uptake on CPET were markedly reduced with 69 ± 13 Watts (35% predicted) and 12 ± 2 mL/min/kg (38% predicted), respectively. All patients showed a typical cardiac limitation pattern and severe ventilatory inefficiency with a slope of ventilation to carbon dioxide output of 42 ± 12. On RHC, patients showed an exercise-induced increase of mean pulmonary artery pressure (from 16 ± 2.4 to 27 ± 2.8 mmHg, p < 0.001), pulmonary artery wedge pressure (from 9 ± 3.3 to 17 ± 5.3 mmHg, p = 0.01), and cardiac output (from 4.7 ± 0.5 to 6.2 ± 1.0 L/min, p = 0.008) with a corresponding slight increase of pulmonary vascular resistance (from 117 ± 35.4 to 125 ± 35.1 dyn*sec*cm^−5^, p = 0.58) and a decrease of mixed venous oxygen saturation (from 58 ± 6 to 32 ± 9%, p < 0.001).

**Conclusion:**

An open aortic valve strategy leads to impaired exercise capacity and hemodynamics, which is not reflected by NYHA-class. Unknown compensatory mechanisms can be suspected. Further studies comparing higher vs. lower support are needed for optimization of LVAD adjustment strategies.

## Background

Left ventricular assist devices (LVAD) are a proved bridge-to-transplantation, and are increasingly utilized as long term support devices for destination therapy [1,2]. Newer generation continuous flow devices (cfLVAD) are characterized by increased durability and suitability for longer support intervals [3]. Currently, there is a debate about the appropriate degree of support. In theory, the maximum achievable support is best as it not only leads to hemodynamic stability, but also increases cardiopulmonary performance. However, full support enhances left ventricular unloading causing presumably more blood damage, dangerous negative intraventricular pressures and hazardous support limitations such as pump stops with an increased risk of thrombus formation. In addition, maximum support increases the pressure over the aortic valve aggravating the development of aortic valve closure, leaflet fusion, valve thrombosis or/and ultimately aortic regurgitation, which seems to be a particular problem in patients with a closed aortic valve on long term support [4-6]. Therefore it seems advisable to perform serial echocardiographic exams to optimize pump speed with regard to normal aortic valve function [7]. The hemodynamic and exercise capabilities following this speed approach are unclear. The literature of cardiopulmonary exercise testing (CPET) and right heart catheterization (RHC) studies during exercise on support is limited [8,9], and the majority of these studies focused on the exercise capabilities of LVAD patients without emphasizing a normal aortic valve function. To our knowledge, no data exists describing exercise performance of patients with optimized pump speed with regard to a normal or near normal aortic valve physiology on long-term support (>6 months). We investigated the hemodynamic profile and exercise capacity in LVAD patients who received pump speed optimization following the mentioned approach.

## Methods

### Patients

Starting January 2009, all consecutive patients who underwent LVAD implantation with the intention to bridge them to transplantation were eligible for the study. As a meaningful comparison of different LVAD systems is impossible, we included only patients with the Berlin Heart INCOR® (Berlin Heart, Germany) operated at a rotor speed according to a normal aortic valve function. A second inclusion criterion was a support time of 6 months without any major adverse event, and the ability to perform CPET.

The routine postimplantation protocol at our institution consists of monthly clinical examinations including the driveline exit site, echocardiographic exam and a cycle ergometry, as well as standard laboratory tests including hemolytic parameters. Our institution recently expanded the testing protocol by including CPET and a sequential routine RHC at rest and on exercise. Data were retrospectively analyzed and entered into a database maintained within the section of Cardiac Surgery. Informed consent for CPET and RHC as well as data collection and anonymized reporting was obtained. Since right heart catheterization belongs to our routine examinations on LVAD support and due to the retrospective nature of the study, formal approval by the institutional review board was waived.

### LVAD system, implantation and speed adjustment

All patients were provided with an INCOR® LVAD system (Berlin Heart, Berlin, Germany), which is not available in the US but widely available in Europe and Asia. The pump is equipped with silicone conduits and a 200 g titanium housing with an outer diameter of 30 mm containing an impeller with magnetic levitation that avoids friction and wear. The rotor spins at 5,000 to 10,000 rpm and provides a flow between 3 and 10 liters per minute. LVAD implantation was performed on a beating heart via median sternotomy, and with the support of extracorporeal circulation. Cardiopulmonary bypass was instituted with cannulation of the distal ascending aorta and the right atrial appendage. The left ventricular apex was elevated and a circular hole with a coring knife were created. After careful inspection of the left cavity 10 to 12 sutures reinforced by a Teflon felt were placed around the access site. The sutures were led through a Dacron ring, which was then connected to the left ventricular apex. The inflow conduit was inserted and fixed to the previously sutured Dacron ring. The pump housing was connected and placed inside the pericardial cavity above the heart, and the drive-line was placed to exit the abdominal wall on the right upper quadrant. The silicone outflow conduit was cut to an appropriate length and sutured end-to-side to the ascending aorta with the help of a vascular side-biting clamp. The pump was started after careful de-airing, and adjusted to provide a pump flow of 4 to 5 L/min, usually at 6,000 to 7,000 rpm. All silicone conduits are only available in one size, the length of the conduits are modifiable according to the anatomy of the patient.

Pump speed was optimized by monthly echocardiographic monitoring with the aim to maintain an aortic valve opening at least once every three heart beats without any septal shifting at rest. In addition, patients should be in NYHA class II and able to perform a sitting cycle ergometer exercise of at least 10 minutes at a workload of 20 Watts.

### Cardiopulmonary exercise testing

We applied a standard CPET protocol on a sitting cycle ergometer (Ergoselect 200, Ergoline, Bitz, Germany) with breath-by-breath gas exchange measurements (MasterScreen™ CPX, Care Fusion, Hoechberg, Germany) and continuous monitoring of 12-lead ECG and oxygen saturation by pulse oxymetry. Capillary blood was used for lactate and gas analysis at rest and maximal exercise. Due to the markedly reduced pulsatile blood flow in the majority of patients, blood pressure measurements were not carried out routinely by the means of ultrasound in a systematic fashion. After 3 minutes of rest and 3 minutes of unloaded cycling, workload was increased by 1 Watt every 6 seconds, resulting in a ramp of 10 Watts/minute. Exercise was symptom-limited or stopped after objective withdrawal criteria were met. Exercise limiting symptoms and dyspnea according to a 10-point Borg scale were recorded. Breath-by-breath data were averaged every 8 breaths and displayed according to Wasserman. The anaerobic threshold was determined by the V-slope method.

### Right heart catheterization

RHC was performed the day after CPET or at the same day after at least 2 hours of resting on an outpatient basis. Patients were placed in a semi-recumbent position on a lying cycle ergometer (Ergo-metrics 900, Ergoline, Bitz, Germany). The pressure transducer was zeroed at 40% of the anterior-posterior chest diameter. The 7 French Swan-Ganz-catheter (Edwards Lifesciences, Irvine, CA, USA) was introduced via a cubital vein (8.5 French sheath, Arrow, Reading, PA, USA) and forwarded into the pulmonary artery by observation of the typical pressure loops. Only in difficult cases fluoroscopy was used for catheter placement. We recorded mean right atrial pressure, systolic and end-diastolic right ventricular pressure, systolic and diastolic pulmonary artery pressure (PAP), and pulmonary artery wedge pressure (PAWP) at the end of a patient’s normal expiration without using breath-hold commands. Cardiac output was obtained by thermodilution averaging three measurements with a maximum deviation of 10%. Measurements were carried out after at least 10 minutes of rest after the catheter placement and at a constant work rate of 20 Watts after PAPs and heart rate stabilized. Pulmonary vascular resistance (PVR) was calculated as (mean PAP-PAWP)/cardiac output × 80 [dyn*s*cm^−5^]. Mixed venous oxygen saturation was determined by blood gas analysis from the pulmonary artery at rest and the end of steady state exercise.

### Statistical analysis

Results are expressed as means ± standard deviations. The normality of distribution of quantitative variables was verified with a Kolgomorov-Smirnov test. Comparisons of baseline data with exercise data were performed using the t-test for paired values. Statistical significance was defined at a p < 0.05. All analyses were performed using SPSS 17 software (SPSS Inc, Chicago, IL).

## Results

### Patient characteristics

Since January 2009, 45 consecutive patients received an LVAD at our institution, and 13 patients were successfully bridged to transplant. After the amendment of our postimplantation monitoring program in January 2012 including CPET and RHC, 21 consecutive patients were seen on an outpatient basis. After exclusion of 11 patients (4 patients other LVAD device, 7 patients unable to perform exercise testing), 10 patients were eligible for the study. Of these, one patient did not provide consent for the invasive procedures, and in one patient RHC was not possible due to difficult vascular access. The remaining 8 patients had a mean age of 45 ± 13 years with a mean support time of 465 ± 257 days at the time of exercise testing, and a mean pump support of 4.0 ± 0.3 l/min at a speed of 6800–7100 rpm. The underlying diagnoses were dilative cardiomyopathy in 6 patients and ischemic cardiomyopathy in 2 patients. All included patients were on an outpatient basis in NYHA class 2. Echocardiography on support at the time of exercise testing showed a mean left ventricular ejection fraction (according to Simpson) of 29 ± 11%, and aortic valve opening was present at least once every three heart beats in all patients at rest. All but one patient were in sinus rhythm at the time of the study. To date all patients are still alive and in a good general condition, and three patients were transplanted during the study period. Further baseline characteristics are shown in Table 1.

**Table 1:** Demographics

	***LVAD patients (n = 8)***
***General Data***	
**Age, years**	45 ± 13
**Height, cm**	174 ± 6
**Weight, kg**	75 ± 10
**Body mass index, kg/m**^**2**^	24 ± 3
**LVEF, %**	29 ± 11
**Male**	8
***Etiology of heart failure***	
**Dilated cardiomyopathy**	6
**Ischemic cardiomyopathy**	2
***Medical treatment***	
**ACE inhibitors**	7 (88%)
**ß-blockers**	7 (88%)
**Diuretics**	8 (100%)
**Aldosteron antagonists**	8 (100%)
**Angiotensin II antagonists**	2 (25%)
**Digoxin**	3 (38%)
**Warfarin**	8(100%)
**Aspirin**	8(100%)
**Clopidogrel**	4 (50%)
***LVAD support***	
**Duration of LVAD support, days**	465 ± 257
**Pump flow, L/min**	4 ± 0.3

### CPET

All patients underwent CPET without adverse events. After a mean exercise time on CPET of 6.0 ± 1.2 minutes, patients reached 69 ± 13 Watts (35 ± 7% predicted). Subjective reason for exercise limitation was dyspnoea in all patients with a median level on the Borg dyspnea scale of 3 (range 2–4). All patients exercised above the anaerobic threshold reaching respiratory exchange rates on exercise > 1 in all but one patient, and the mean respiratory exchange rate increased further after exercise to a maximum of 1.25 ± 0.11. The lactate concentration increased significantly from 9 ± 2 mg/dL to 40 ± 24 mg/dL (p = 0.006). All patients showed a typical pattern of cardiac limitation. The mean oxygen uptake (VO_2_) at the anaerobic threshold and at peak exercise (Peak VO_2_) was markedly reduced with 7.8 ± 1.3 mL/min/kg (24 ± 6% of predicted peak VO_2_) and 12 ± 2 mL/min/kg (38 ± 8% predicted), respectively. The mean slope of ventilation to carbon dioxide output (VE/VCO_2_-slope) was 42.3 ± 11.7. Further CPET variables are displayed in Table 2.

**Table 2:** CPET

**Parameter**	**Observed**	**% Predicted**
**Max. work load, Watt**	69 ± 14	35 ± 7
**HR at peak exercise, min**^**−1**^	125 ± 24	72 ± 12
**Max. O**_**2**_**-pulse, mL**	8.1 ± 1.5	52 ± 7
**VO**_**2 **_**at AT, mL/min**	631 ± 120	24 ± 6*
**VO**_**2 **_**at AT, mL/min/kg**	7.8 ± 1.3	
**VO**_**2 **_**at peak exercise, mL/min**	1004 ± 233	38 ± 8
**VO**_**2 **_**at peak exercise, mL/min/kg**	12.4 ± 2.5	
**VE/VCO**_**2**_**-slope**	42.3 ± 11.7	-
**BR at peak exercise, %**	49 ± 15	> 20
**Oxygen saturation at rest, %**	97.1 ± 1.3	-
**Oxygen saturation at peak exercise, %**	96.2 ± 1.1	-

* % of predicted Peak-VO_2_.

BR = breathing reserve; HR = heart rate; VCO_2_ = carbon dioxide output; VE = ventilation per minute; VO_2_ = oxygen uptake.

### Right heart catheterization

**Figure 1:**
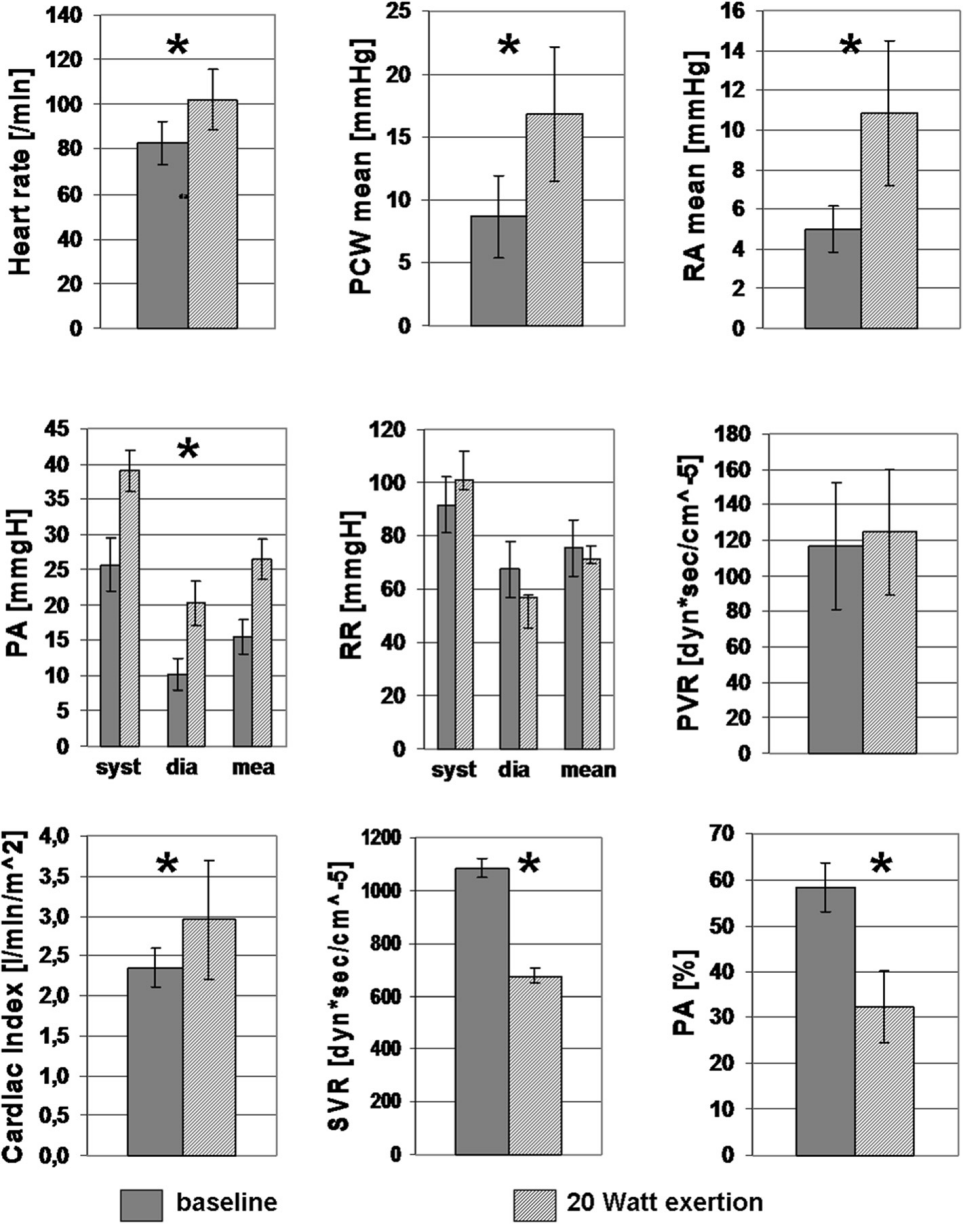
**Synopsis of the results.** Abbr.: PCW = pulmonary capillary wedge pressure; RA = right atrial pressure; PA [mmHg] = pulmonary artery pressure; RR = blood pressure (in 2 patients), PVR = pulmonary vascular resistance; SVR = systemic vascular resistance, PA [%] = mixed venous saturation, * = significant difference from baseline (p < 0.05).

RHC was successful in all patients without adverse events. All patients were able to perform a complete steady state exercise at 20 Watts without the perception of exertion or dyspnoea. Cardiac output increased moderately from 4.7 ± 0.5 L/min to 6.2 ± 1.0 L/min (p = 0.008), and so did heart rate from 83 ± 9.8 beats/min to 102 ± 13.4 beats/min (p = 0.02). All evaluated pressures increased significantly performing the steady state low work load exercise. Right atrial pressure increased from 5 ± 1.1 mmHg to 11 ± 3.6 mmHg (p =0.007), mean PAP increased from 16 ± 2.4 mmHg to 27 ± 2.8 mmHg (p < 0.001), and PAWP increased from 9 ± 3.3 mmHg to 17 ± 5.3 mmHg (p = 0.01). PVR increased slightly from 117 ± 35.4 dyn*sec*cm^−5^ to 125 ± 35.1 dyn*sec*cm^−5^ (p = 0.58). Systemic vascular resistance could be evaluated in two patients, since blood pressure was not reliably measurable in all patients, and in these two patients it decreased from 1084 ± 210.5 dyn*sec*cm^−5^ to 678 ± 26.5 dyn*sec*cm^−5^. Mean mixed venous oxygen saturation decreased significantly from 58 ± 6% to 32 ± 9% (p < 0.001). Further hemodynamics are depicted in Figure 1.

## Discussion

This study was the first evaluation of maximal CPET and invasive hemodynamics under exercise on left ventricular support with a speed adjustment according to aortic valve opening. The results can be summarized as follows: The maximum work load on CPET was approximately 70 Watts which is roughly 35% of predicted. Correspondingly, the mean peak VO_2_ was markedly reduced. Importantly, patients exercised until exhaustion as reflected by the increase in arterial lactate concentration and respiratory exchange ratio. Interestingly, all patients were able to perform a complete steady state exercise at 20 Watts without the perception of exertion or dyspnoea. However, given the very low work rate securely below the anaerobic threshold corresponding to approximately 35% of predicted maximal work rates, increases in PAWP and PAP are clearly above the physiologic response reflecting left ventricular failure on exercise. The severity of cardiac impairment is also reflected by the only moderate increase in cardiac output and the marked decline in mixed venous oxygen saturation. Of note is also the clinically unapparent but low baseline mixed venous saturation of 58% as a sign of controlled marginal support due to the open aortic valve support policy in this patient cohort. Adaptive and compensatory mechanisms in this chronic heart failure population may explain the clinically unapparent marginal support.

In order to interpret the RHC results under LVAD support properly, a comparison with hemodynamics of healthy subjects needs to be done, since no control group with full support is available. In healthy individuals older than 50 years an increase of PAWP and left heart enddiastolic pressures under exercise seems to be physiologic [10,11], and it is a physiologic behaviour that the PVR slightly decreases under exercise [10]. The LVAD population of this study displayed an increase in PAWP comparable to healthy adults aged older than 50 years [10], but at the same time and different to healthy subjects an increase in PVR. The missing decrease in PVR can be explained partially by irreversible remodelling processes of the pulmonary vascular bed due to long lasting left heart failure, and the marginal support policy of the presented patient population resulting in only a minor increase in cardiac output during exercise [12]. The PAWP increase under exercise was also seen by Maybaum et al. in patients with an ejection fraction of 40% on support and on an outpatient basis with PAWP values of 15 mmHg on an acute reduction of pump flow to 2–3 L/min according to their weaning protocol [13].

Previous reports of exercise capacity in patients with LVADs are mostly from relatively young patients implanted under the concept of bridge to transplantation [8,14]. Leibner et al. monitored the exercise capacity by repetitive peak VO_2_ measurements in a cfLVAD population similar to our cohort before and at certain time points up to 1 year after implantation of cfLVADs. Surprisingly, no statistical improvement in peak VO_2_ at any time point after implantation was noted with a mean peak VO_2_ around 12 ml/min/kg similar to our values. These findings raise the question whether peak VO_2_ measurements offer an appropriate monitoring tool in cfLVAD patients, since clinically significant improvements are observed. On the other hand, Jakovljevic *et al.* reported an improvement after LVAD implantation with a peak VO_2_ of 19.8 ± 5.8 ml/min/kg for patients supported with cfLVAD at the Harefield Hospital in the UK, which is much higher than that measured in our patient population. This discrepancy can be explained by a younger age of the population (39 y vs. 45 y), in which LVAD weaning was considered and a high left ventricular ejection of 50% compared to 30% in our patient population [15,16]. Jacquet et al. reported a maximum workload sustained by cfLVADs of 68 Watts with a peak VO_2_ of 15.8 mL/min/kg at a pump flow of 5.2 L/min. [17]. Mancini et al. found similar values for peak VO_2_ and peak exercise in pulsatile devices [18]. Haft et al. showed that pulsatile and continuous flow LVADs lead to similar exercise capacities at higher flows compared to our study population, demonstrating peak VO_2_ of approximately 15 mL/min/kg [8].

RHC still built the gold standard to accurately evaluate hemodynamics. However, RHC is invasive with a risk of adverse events explaining the scarcity of RHC on LVAD support in general and under exercise even more. We decided to include RHC utilizing the cubital vein without discontinuation of anticoagulation at least once into our routine post LVAD-implantation protocol for reliable measurement of cardiac output and more important PAPs, since pump speed is opted to be as low as possible for several reasons. The main reason is a normal physiology of the aortic valve to prevent deleterious aortic regurgitation or thrombus formation [19]. Other reasons to adjust pump speed to a an opening aortic valve are a decrease of the likelihood of suction alarms by avoiding full emptying of the left ventricle resulting in less blood trauma due to reduced sheer stress [20]. Therefore, our institutional policy is a rather low pump support (e.g. 4 L/min in the presented patient cohort). This policy seems to be of growing interest, since devices are more and more used in aged patients as destination therapy or for longer periods of time [5,21,22]. Also partial support devices seem to be a real alternative to the existing full support devices on the market [20,23]. This strategy is supported by similar CPET capacities under this submaximal support and an open aortic valve policy compared to previous invasive exercise studies of LVAD patients on higher LVAD support levels. Remarkably, although exercise capacity and hemodynamics were markedly impaired, the patient population of this study was clinically stable in NYHA 2 on an out-patient basis. However, the effects of this submaximal support policy on long-term outcome are yet unclear.

### Limitations

There are certain limitations associated to this study. First of all, a single institutional experience is reported in a retrospective manner, and only one device in a small but homogeneous group of patients was tested. Other pumps might have a different impact on hemodynamics during exercise, because of a different preload and afterload behaviour. However, our institution mainly implants the INCOR® Berlin Heart, therefore we included only patients with this device to exclude device related impacts on exercise performance. The next step will be to systematically evaluate hemodynamic consequences of exercise in other devices (e.g. centrifugal pumps vs. axial pumps). Further, the near normal functioning of the aortic valve was only checked on routine echocardiograms every 4 weeks and not immediately before and during exercise.

No control group was tested to see whether higher support levels lead to higher exercise capacities or improved hemodynamics. There are several reasons for this. First, our open aortic valve policy applies to all of our LVAD patients. Therefore, a control population can’t be generated out of our institution. The same population could be a control with higher pump flows. However, we decided not to set the pump speed higher and to perform the same exercise test, since patients were clinically stable and setting the pump speed higher leads to significant more suction alarms, which we wanted to avoid in this outpatient population. A systematic blood pressure measurement was not performed since ultrasound was not available for each included patient. In addition simultaneous pump flow measurements in order to evaluate native cardiac function more appropriate is missing. However, since the remaining cardiac function interacts with pump flow detailed measurements of flows on LVAD were not performed.

## Conclusion

The presented study has shown that a submaximal LVAD support in order to keep a near physiologic aortic valve function seems to be feasible with regard to “clinical” exercise capacity as reflected by NYHA-class. However, maximal exercise capacity and hemodynamics under submaximal LVAD support are still significantly impaired. Adaptive mechanisms in long term LVAD support seem to compensate submaximal support leading to clinically less impaired exercise capacity. These mechanisms are unclear and not explained by this study. Further studies are necessary to reveal them and to determine the impact of our findings on long-term outcome.

## Abbreviations

cfLVAD: Continuous flow left ventricular assist device; L/min: Liters per minute; CPET: Maximal cardiopulmonary exercise testing; RHC: Right heart catheterization; NYHA class: New York Heart Association functional classification; VO_2_: Oxygen uptake; PVR: Pulmonary vascular resistance; PAWP: Pulmonary artery wedge pressure; PAP: Pulmonary artery pressure; RPM: Rounds per minute.

## Competing interests

The authors declare that they have no competing interests.

## Authors’ contributions

DC, data collection, exercise testing, and presentation of the data as text and writing. TJL, data collection, exercise testing and presentation of the data as text and writing. PG, Data aqusition, Echocardiography, patient care, proof reading of the manuscript. SH, patient care, helped to draft the manuscript. BF, Echocardiography, patient care, proof reading of the manuscript. YZ, Anesthesia, helped to draft the manuscript. LR, Implanting surgeon, patient care, helped to draft the manuscript. MH, Implanting surgeon, patient care, helped to draft the manuscript. CS, Implanting surgeon, patient care, participated in the design of the study, helped to draft the manuscript, correction of the manuscript, comments to the reviewers. All authors read and approved the final manuscript.
